# SOCIAL COHESION AND ORAL HEALTH PROBLEMS AMONG U.S. OLDER CHINESE ADULTS

**DOI:** 10.1093/geroni/igz038.1268

**Published:** 2019-11-08

**Authors:** Weiyu Mao, Weiyu Mao, Bei Wu, Iris Chi, Wei Yang, XinQi Dong

**Affiliations:** 1 University of Nevada, Reno, Reno, Nevada, United States; 2 New York University, New York, New York, United States; 3 University of Southern California, Los Angeles, California, United States; 4 Rutgers University, New Brunswick, New Jersey, United States

## Abstract

This study examined the relationship between social cohesion (i.e., sense of community and neighborhood cohesion) and self-reported number of oral health problems and further investigated the potential moderating role of cognitive function in such a relationship among U.S. older Chinese adults. Data came from baseline of the Population Study of Chinese Elderly in Chicago between 2011 and 2013 (N = 3,157). Stepwise negative binomial regression models with interaction terms were used. Individuals with a stronger sense of community had 1% less risk of having oral health problems (RR = .99; 95% CI = .98, .99; p < .001). Individuals experiencing a stronger neighborhood cohesion had a 11% reduction in risk of having oral health problems (RR = .89; 95% CI = .86, .92; p < .001). To promote optimal oral health, interventions need to account for individuals’ perception and actual integration with their neighborhood and communities.

